# Introduction of customized inserts for streamlined assembly and optimization of BioBrick synthetic genetic circuits

**DOI:** 10.1186/1754-1611-4-17

**Published:** 2010-12-20

**Authors:** Julie E Norville, Ratmir Derda, Saurabh Gupta, Kelly A Drinkwater, Angela M Belcher, Andres E Leschziner, Thomas F Knight

**Affiliations:** 1 Computer Science and Artificial Intelligence Laboratory, Massachusetts Institute of Technology, Cambridge, MA 02139, USA; 2 Biological Engineering Division, Massachusetts Institute of Technology, Cambridge, MA 02139, USA; 3 Department of Materials Science and Engineering, Massachusetts Institute of Technology, Cambridge, MA 02139, USA; 4 Department of Chemistry, University of Alberta, Edmonton, Alberta T6G 2G2, Canada; 5 Department of Chemistry and Chemical Biology, Harvard University, Cambridge, MA 02138, USA; 6 Wyss Institute for Biologically Inspired Engineering, Harvard University, Cambridge, MA 02138, USA; 7 Department of Molecular and Cellular Biology, Harvard University, Cambridge, MA 02138, USA; 8 Ginkgo BioWorks, 7 Tide St., Unit 2B, Boston, MA 02210, USA

## Abstract

**Background:**

BioBrick standard biological parts are designed to make biological systems easier to engineer (e.g. assemble, manipulate, and modify). There are over 5,000 parts available in the Registry of Standard Biological Parts that can be easily assembled into genetic circuits using a standard assembly technique. The standardization of the assembly technique has allowed for wide distribution to a large number of users -- the parts are reusable and interchangeable during the assembly process. The standard assembly process, however, has some limitations. In particular it does not allow for modification of already assembled biological circuits, addition of protein tags to pre-existing BioBrick parts, or addition of non-BioBrick parts to assemblies.

**Results:**

In this paper we describe a simple technique for rapid generation of synthetic biological circuits using introduction of customized inserts. We demonstrate its use in *Escherichia coli *(*E. coli*) to express green fluorescent protein (GFP) at pre-calculated relative levels and to add an N-terminal tag to GFP. The technique uses a new BioBrick part (called a BioScaffold) that can be inserted into cloning vectors and excised from them to leave a gap into which other DNA elements can be placed. The removal of the BioScaffold is performed by a Type IIB restriction enzyme (REase) that recognizes the BioScaffold but cuts into the surrounding sequences; therefore, the placement and removal of the BioScaffold allows the creation of seamless connections between arbitrary DNA sequences in cloning vectors. The BioScaffold contains a built-in red fluorescent protein (RFP) reporter; successful insertion of the BioScaffold is, thus, accompanied by gain of red fluorescence and its removal is manifested by disappearance of the red fluorescence.

**Conclusions:**

The ability to perform targeted modifications of existing BioBrick circuits with BioScaffolds (1) simplifies and speeds up the iterative design-build-test process through direct reuse of existing circuits, (2) allows incorporation of sequences incompatible with BioBrick assembly into BioBrick circuits (3) removes scar sequences between standard biological parts, and (4) provides a route to adapt synthetic biology innovations to BioBrick assembly through the creation of new parts rather than new assembly standards or parts collections.

## Background

In traditional modification of organisms by cloning [1], the emphasis has been on single gene changes that improve the organism or make a single component easier to study. Construction of synthetic genetic circuits brings together many components [2,3] to accomplish novel tasks, creating functions unobtainable through single gene changes. *De novo *construction of genetic circuits encompasses the techniques that fall into two categories: techniques for construction and techniques for optimization. Gene synthesis, though decreasing in price [4], still remains prohibitively expensive for *de novo *synthesis of complete genetic circuits [5]. Instead, either newly synthesized, natural, or existing DNA fragments are pieced together using DNA assembly techniques. A variety of assembly methods now exist including idempotent methods [5-12], extensions to idempotent methods [13-18], ligation independent methods [3,19-21], USER enzyme based methods [22,23], multi-part enzymatic assembly methods [24,25], and genome-scale assembly methods [26-28]; however, regardless of how genetic circuits are constructed, novel circuits almost always require modification and optimization. Because understanding of the relevant biological mechanisms remains incomplete [29], one of the main problems in newly assembled circuits is mismatch in the expression levels of the components of the circuit [30]. Optimization, thus, involves modifications of the expression levels [4,31] to increase desired products, decrease toxic by-products, and increase limiting reagents [32-34]. A number of existing methods could be used to optimize circuits by rebuilding [29] or reengineering [21,35] them. It is desired, however, to minimize the number of steps and permit rapid modification [35-41].

**Figure 1:**
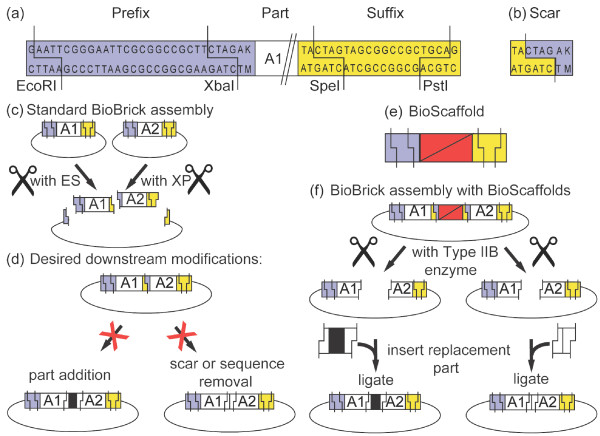
**Desired BioBrick circuit modifications and approach with BioScaffolds**. Every BioBrick standard biological part **(a) **consists of a DNA sequence embedded between a "prefix" sequence (purple box) and a "suffix" sequence (yellow box). Parts may also contain scars **(b)**, which form when two parts, such as "A1" and "A2" in **(c) **are fused together using BioBrick assembly [12]. In many cases one would like to convert an undesired scar between two parts in a BioBrick assembly into a different part or completely remove it **(d)**. Our approach is to create a new BioBrick part (the BioScaffold) **(e)**. The BioScaffold can be assembled into a circuit using BioBrick assembly, but unlike normal BioBricks it can be removed and replaced with a new part **(f)**. In this paper we develop a single prototype BioScaffold that illustrates how BioScaffolds can be used to either insert parts or remove scars.

BioBrick assembly constitutes a widely used strategy for assembly of custom genetic circuits [5]. BioBrick parts are DNA pieces with standard sticky ends (Figure 1a and 1c). Using BioBricks, large circuits can be rapidly assembled using a sequence of similar steps. Over 5,000 BioBrick standard biological parts are freely available to researchers through the Registry of Standard Biological Parts [42]. Although BioBricks have been used to construct a large variety of genetic circuits [12,43-54], these circuits often require optimization [44,55-59] and currently, there is no standard methodology for optimizing BioBrick circuits.

Modification of a circuit's ribosome binding sites (RBSs) is an attractive method for optimization since different strength RBSs create large changes in circuit behavior [35] and a web-based tool is now available to design RBSs of different strengths [36]. Inserting regulatory regions with predetermined sequences changes the levels of protein expression [35,36] and can have dramatic effects on circuit performance. Optimizing BioBrick circuits using modification of the RBS, however, requires overcoming a fundamental limitation of the BioBrick assembly method: it does not provide a way to insert parts into the circuits once they are assembled (Figure 1d). In this paper, we demonstrate a solution to this problem by designing a new BioBrick part, termed BioScaffold (Figure 1e and 1f), that can be easily excised from intact BioBrick circuits and replaced with other DNA sequences (e.g., RBSs). By virtue of its design, BioScaffolds also bypass two other fundamental limitations of BioBrick assembly: (1) it allows incorporation of parts that contain the sites recognized by the enzymes EcoRI, XbaI, SpeI, and PstI (these sites are incompatible with standard BioBrick assembly) and (2) it allows removal of a scar site (Figure 1b and 1f) with sequence TACTAGAK (where K = G or T [60]) between BioBrick parts (this is useful because the presence of the stop codon sequence TAG in the scar interferes with production of protein fusions and other modifications [11].)

To outline the design of the BioScaffold part, we first review a hypothetic circuit built from components "A1" and "A2" (designated here as "A1-scar-A2"). Removal of the scar sequence or its replacement with a custom regulatory element (e.g., a RBS) can be performed in two steps. In the first step, excision of the scar and small regions of "A1" and "A2" leaves "sticky ends" inside "A1" and "A2." These ends can then be used for the ligation of the opening with a scar-less DNA sequence or a short sequence that contains a custom ribosome binding sequence. For example, if the excised region is (10 bp of A1)-scar-(12 bp of A2), ligating the gap with 22 base pair annealed repair oligonucleotides consisting of (10 bp A1)-(12 bp A2) forms a scar-less sequence "A1-A2." While the second step is easily accomplished, the first step requires a specialized enzyme that recognizes scar sequence TACTAGAK and cuts an arbitrary 10 base pairs to the left and 12 base pairs to the right of it. Unfortunately, no enzymes that bind to the sequence TACTAGAK and cut on both sides of the sequence (but outside of it) are available at this time. REases that cleave outside their recognition sites are known [61-64], but none satisfy the specific requirements of this application. The evolution of an enzyme that can excise the scar sequence TACTAGAK is in principle possible [65-67], but not trivial.

**Figure 2:**
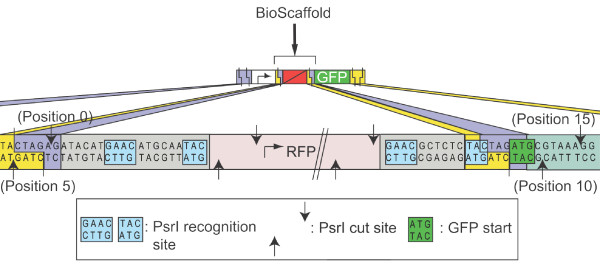
**The prototype BioScaffold {0,5;15,10} embedded in the test circuit**. Here, the internal structure of the prototype BioScaffold and the locations of the associated PsrI cut sites and recognition sequences are shown. The prototype BioScaffold (part BBa_J70399 in the Registry of Standard Biological Parts) is assembled between a promoter (BBa_R0010, which is not shown here) and GFP (BBa_E0040, which is partially shown here). The BioScaffold contains one PsrI recognition site on either side of the RFP reporter (as well as an internal PsrI site within the RFP reporter that is not shown). PsrI cuts into the scar on the left side of the part and GFP on the right side of the part, allowing the BioScaffold to be replaced with RBS sequences that control the expression of GFP. When the BioScaffold is present, its internal RFP reporter circuit is also present and should produce red fluorescent colonies. The RFP reporter circuit should be removed when the BioScaffold is removed. The cutting profile for the BioScaffold is {0,5;15,10}, using the notation {w,x;y,z} is described in the Results section of the paper. Different BioScaffolds can be created with different restriction enzyme sites, reporters, or cutting profiles.

**Figure 3:**
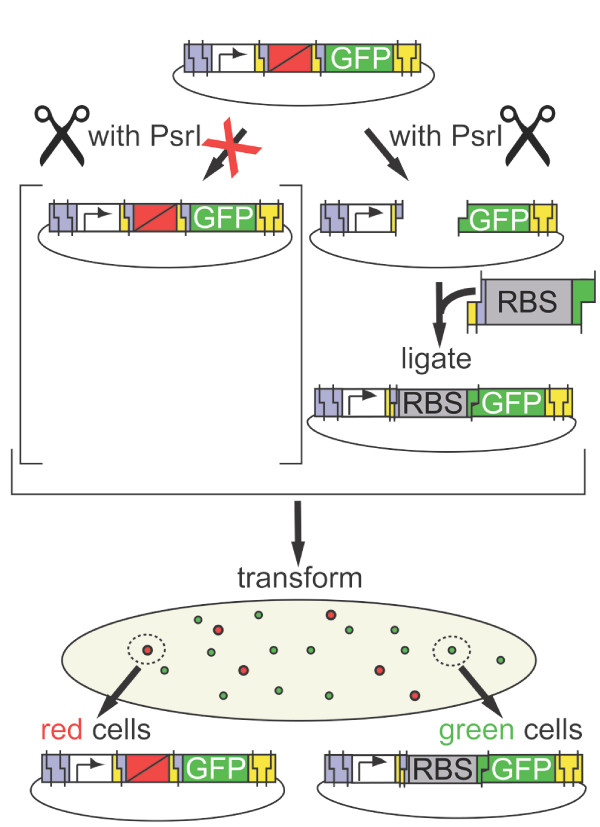
**Excision and selection for BioScaffold removal**. Here, the prototype BioScaffold is present in a test circuit, with sequence "promoter-scar-BioScaffold part-scar-GFP." It is cut with the enzyme PsrI and then replaced with a RBS sequence. The BioScaffold test part contains a RFP expression circuit surrounded by two PsrI recognition sites; thus, cells that contain the BioScaffold part, such as the cells that contain the test circuit, exhibit red fluorescence. If PsrI excises the BioScaffold part from the test circuit and an RBS is ligated across the open gap, then the sequence "promoter-scar-RBS-GFP" is obtained. Cells that contain this sequence exhibit green flourescence, where the strength of the fluorescence depends on the RBS used. To show the flexibility of the BioScaffold, it was also replaced with a "RBS-MBP-GS" sequence to create the sequence "promoter-scar-RBS-MBP-GS."

As an alternative, one can use an existing Type IIB or IIS REase that can cleave outside its recognition sequence and modify the "scar region" between parts "A1" and "A2" to introduce the recognition sequence. Additional file 1 Table S1 lists an assortment of Type IIB REases that cleave the target on both sides of their recognition sequence [61,64]. Unfortunately, the cleavage efficiency of most Type IIB REases is low. For example, the efficiencies of ArsI and PsrI are above 56% (e.g., for PsrI more than 70% of DNA fragments can be ligated and 80% of these can be recut), whereas REases commonly used in cloning experiments, such as EcoRI and SpeI, typically have efficiencies above 90% http://www.sibenzyme.com. To facilitate selection of the constructs that will undergo cutting (and subsequent ligation with an arbitrary DNA sequence), we sought to introduce a reporter inside the excised region. Final design of the BioScaffold part, hence, contains two Type IIB recognition sequences placed on either side of RFP reporter (as well as an additional site within the RFP reporter) that serves as a selection marker (Figure 2). This configuration makes it simple to select colonies that circuits in which the BioScaffold has been placed or excised and replaced with repair oligonucleotides (Figure 3).

In this paper we demonstrate the utility of a BioScaffold to optimize BioBrick circuits (by inserting a series of RBS regulatory sequences) as well as for production of protein fusions. Because none of these processes can be easily attained through use of standard BioBrick assembly, these results demonstrate that the use of BioScaffolds can aid in overcoming several limitations of BioBrick assembly.

## Results

### Maximum excision capacity of commercially available Type IIB REases

As described in the preceding section, the prototype BioScaffold is primarily useful for introducing RBSs (Circuit Tuning BioScaffold) or N-terminal protein tags (Protein Engineering BioScaffold) into BioBrick circuits that do not contain internal PsrI restriction enzyme recognition sites or RFP reporters. Thus, a variety of BioScaffolds might be desirable for other applications or for use with BioBrick circuits that contain PsrI sites or RFP reporters. To provide a sense of the extendibility of BioScaffolds, we examined a variety of restriction enzymes that can cut outside their restriction enzyme recognition sites and can thus be used to create the cloning site within a BioScaffold. For several commercially available Type IIB enzymes we have determined the maximum number of nucleotides from the surrounding parts "A1" and "A2" that can be excised using an intermediate BioScaffold inserted between them. To find the maximum number of excised nucleotides for each enzyme, we align the enzyme recognition sites to the outermost position where they can bind to the left and right scar sequences. The resulting alignments, shown in Additional file 1 Table S1, demonstrate that the furthest distances the enzymes can cut into left part "A1" and the right part "A2" are 9 nucleotides into "A1" for enzyme BaeI and 3 nucleotides (plus 2 additional nucleotides for downstream protein parts) into "A2" for enzymes BaeI and CspCI. When part "A2" is a protein coding region then the last 2 nucleotides from the scar region are included in part "A2," since the last two nucleotides of the scar TACTAGAK become the first 2 nucleotides of the start codon ATG.

### BioScaffold notation, {w,x;y,z}

Since other BioScaffolds beyond the circuit tuning and N-terminal protein engineering application (or for the same function (tuning circuits) where PsrI restriction enzyme sites or RFP reporters are present) might be desired, we created a notation to make it simpler to compare two BioScaffolds. We define the following notation {w,x;y,z} to describe in condensed form the positions of the cut sites relative to the BioScaffold: w is the position of the left cut on the forward strand, x is the position of the left cut on the reverse stand, y is the position of the right cut on the forward strand, and z is the position of right cut on the reverse strand. The position numbering begins on the right side of the BioScaffold part with 0 as the first position before TACTAGAK, where numbers increase to the right, and on the left side with -1 as the first position after the K of TACTAGAK, where numbers increase to the left--see Additional file 1 Table S1 for examples. The cut sites of the prototype BioScaffold are given by {0,5;15,10} (see Figure 2 where the excision positions are marked above the cut sites). When referring to a BioScaffold in BioBrick format, the notation {w,x;y,z} assumes that the BioScaffold is in its most common form when used with BioBricks (i.e., surrounded by a scar sequence on either side).

### Testing the BioScaffold

**Figure 4:**
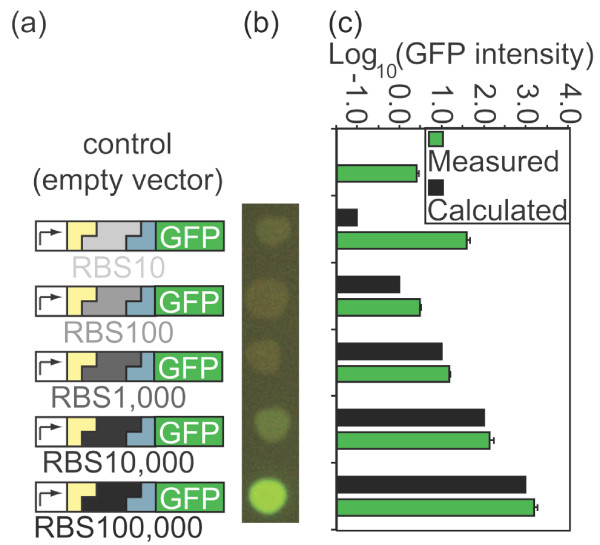
**GFP expression levels created by the different RBSs**. Sequence verified clones of the form "promoter-scar-RBS-GFP" **(a) **were analyzed visually **(b) **and using flow cytometry **(c)**. The inserted RBSs were expected to drive relative translation initiation rates of 10, 100, 1,000, 10,000, and 100,000 for the downstream sequence GFP with the cell fluorescence proportional to the translation initiation rate **(a)**. Qualitative visual assessment revealed green fluorescent intensity commensurate with the expected values **(b)**, although colony thickness can influence perceived intensity. Simultaneously transformed colonies of RBS10, RBS100, and RBS1,000 appeared light green, while RBS10,000 appeared green and RBS100,000 appeared bright green **(b)**. Quantitative assessment using flow cytometry data revealed GFP intensity levels commensurate with expected values, except for the higher than expected translation initiation of GFP driven by RBS10 **(c)**.

**Figure 5:**
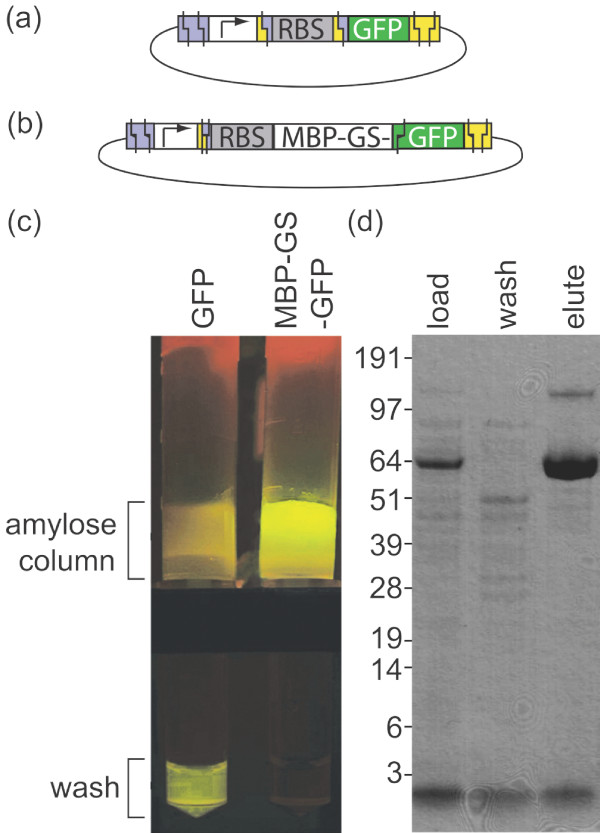
**Properties of GFP with an N-terminal MBP-GS fusion**. Lysate supernatants from cultures expressing GFP alone **(a) **or the MBP-GS-GFP fusion **(b) **were applied to amylose columns. The green fluorescent product was not retained on the "GFP" column when column buffer was applied, but rather appeared in the first wash eluate **(c)**. In contrast the green fluorescent product was retained on the "MBP-GS-GFP" column when column buffer was applied **(c)**. The protein purification process for MBP-GS-GFP was monitored using SDS-PAGE: Lane 1, cell lysate ("load"); Lane 2, first wash eluate ("wash"); Lane 3, maltose eluate ("elute") **(d)**. Mass spectroscopy confirmed that the band at ~67 kDa was MBP-GS-GFP and identified the minor contaminant at the top of Lane 3 ("elute") as a dimer form. The dimer is known to be a normal occurrence with this construct [69,70].

As a demonstration, we positioned the prototype BioScaffold in a synthetic circuit between a promoter and GFP (Figure 3). Replacement of the BioScaffold with an RBS caused this test circuit to become a GFP reporter (containing a promoter, RBS, and GFP) that expresses GFP within the cell (Figure 4). Alternatively, replacement of the BioScaffold with a RBS-(maltose-binding protein)-(glycine-serine) (RBS-MBP-GS) sequence created a circuit that produced a MBP-GS-GFP fusion protein (Figure 5) that is fluorescent green and binds to amylose resin. The prototype BioScaffold has been designed to contain specific cut locations on either side of its sequence (Figure 2). After excision (Figure 3), the BioScaffold will be replaced with one of several RBSs that are designed to drive well-defined levels of expression in the downstream gene, demonstrating how BioScaffolds can be used to facilitate the optimization of circuits. Alternatively, replacement of the BioScaffold with the MBP-GS fusion protein part will demonstrate how BioScaffolds can be used to create protein fusions. The visual markers used in the prototype system help track the presence of the BioScaffold (which contains an RFP reporter) and the performance of the optimization process (effect of different strength RBSs or a MBP-GS fusion on the GFP reporter). Figure 3 shows graphically the proposed replacement of the BioScaffold with repair oligonucleotides that contain an RBS sequence, which will affect the performance of the final GFP reporter circuit (Figure 4). Figure 5 shows that the BioScaffold can successfully be replaced with an expressed MBP-GS fusion, which causes the final protein fusion to gain the property of affinity to an amylose column as well as maintaining the fluorescence of GFP.

### Excision of the BioScaffold and replacement with the RBS and the RBS-MBP-GS sequences

Excision of the BioScaffold from the "promoter-scar-BioScaffold-scar-GFP" composite part and ligation of the RBS insert was expected to create a "promoter- scar-RBS-GFP" composite (where one RBS was inserted in each of five parallel reactions). The plated transformations of each reaction contained a mixture of red colonies and green colonies. It was assumed that the red colonies resulted from clones where the BioScaffold was not excised. At the optimal concentration of vector in the cutting reaction, less than 3% per 1,000 colonies were red (i.e., still contained the BioScaffold.) We picked 25 non-red clones for sequencing (5 for each RBS insert). For 80% of the non-red clones, sequencing showed that the RBS was inserted in place of the BioScaffold part at the correct location with the correct sequence. For 12% of the clones, sequencing showed that the entire test circuit was completely mutated out. For 8% of the clones, the sequencing result was noisy, preventing interpretation. We speculate that none of the errors are due to the cutting properties of the enzyme, but rather to selection against the circuit or inadvertent picking of multiple colonies. Using the same starting circuit, selection, and circuit verification protocol, ligation of the RBS-MBP-GS insert was expected to create a "promoter-scar-MBP-GS-GFP" circuit where the ATG start codon of the GFP was removed by the BioScaffold.

### Expression levels of the RBSs

Colony and sequence verified clones that contain RBSs in place of the BioScaffold part were assessed qualitatively and quantitatively (Figure 4). Visual analysis demonstrated that the clones exhibited a range of green fluorescent intensities: the lowest predicted expression levels appeared light green and the highest expression levels appeared bright green (Figure 4). Visual analysis is only a rough guide to intensity since the number of cells present in a colony could influence its appearance. Overall, the flow cytometry data confirmed the results from [36] using our method to introduce the RBS. Flow cytometry data indicated that the predicted expression levels were commensurate with the actual intensity values except for sequence RBS10, which was expected to produce the lowest intensity of protein fluorescence (Figure 4). The actual expression level for RBS10 was higher than expected; however, similar deviations are observed by Salis, et al. [36] for low RBS translation initiation strengths, and therefore appear to be unrelated to the use of the BioScaffolds. If it was important to create a circuit with low expression of GFP, we could have performed another optimization round using RBSs with similar but slightly different relative translation rates, such as 8, 9, 11, and 12.

### Affinity of the MBP-GS-GFP protein to an amylose column

The following observation confirmed the presence of desired genetic product (MBP-GS-GFP) in the cells that contained the "promoter-scar-RBS-MBP-GS-GFP" circuit (Figure 5a): (1) cells which contained MBP-GS-GFP exhibited strong fluorescence similar to those of cells that contained the classic BBa_J04430 GFP reporter circuit [68] (Figure 5b), (2) running a lysate from a culture of MBP-GS-GFP cells through an amylose column led to retention of the fluorescent product, which could only be eluted with a buffer containing maltose, whereas for GFP cells the fluorescent product was eluted in the wash (Figure 5c) and (3) both crude MBP-GS-GFP lysate and eluate from the amylose column contained a strong band at ~67 kDa and a weak band at ~134 kDa in a SDS page gel (Figure 5d, "LOAD" and "ELUTE" lanes), which are the expected sizes of a MBP-GS-GFP fusion and a MBP-GS-GFP dimer [69,70], rather than the 40 kDa length of MBP-GS or the 27 kDa length of GFP. The identity of the bands was confirmed by mass spectrometry. (The concentric patterns seen on the gel are artifacts that come from the glass of the scanner.)

## Discussion

### Use of the BioScaffold test circuit to overcome limitations in BioBrick assembly and comparison with other methods

The original protocol for BioBrick assembly does not allow insertion of parts into premade assemblies and optimization of the performance of premade circuits. We demonstrate that BioScaffolds enable optimization of premade circuits through insertion of RBSs of predetermined strength and protein fusions. Although a few other approaches have been recently developed for the same purpose (see below), the use of BioBricks provides advantages and flexibility not present in these methods. For example, Church and co-workers demonstrated rapid evolution of circuits with RBSs using Multiplex Automated Genome Engineering (MAGE) to create and select for circuits with improved performance [35]. Sauro and co-workers used the Clontech In-Fusion PCR Cloning kit to re-engineer BioBrick circuits [21] after assembly. Substitution of the BioScaffold with RBSs provides an approach different from MAGE and In-Fusion because the expression of proteins was manipulated rationally using the RBS calculator [36]. In addition, BioScaffolds can be used to insert both long and short sequences.

BioBrick parts cannot contain restriction sites for EcoRI, SpeI, XbaI, and PstI; thus, conversion of arbitrary DNA sequences into BioBrick parts requires the time-consuming and expensive process of removing these sites from the sequence of interest. We demonstrate, however, that this BioScaffold can be used to insert any parts into BioBrick circuits except for those that contain PsrI recognition sites. Importantly, PsrI recognizes GAACNNNNNNTAC (or GTANNNNNNGTTC) and the probability of encountering this sequence in a random DNA sequence (once every 8,192 base pairs for the forward or reverse recognition site) is 8 times lower than that of encountering a site for EcoRI, SpeI, XbaI, or PstI. In the event that the site for PsrI is present in the insert part, it is possible to use another restriction enzyme (see Additional file 1, Table S1) to create appropriate sticky ends for the insert.

## Conclusions

We demonstrated that BioScaffolds could be implemented as a BioBrick part, integrated into BioBrick circuits, and used to remove a scar sequence. Additionally, BioScaffolds allowed the introduction of parts directly into preassembled circuits. This work demonstrated the introduction of RBSs for circuit optimization, but natural sequences, BioBrick parts, or assemblies of parts can alternately be incorporated. Specifically, appropriately designed BioScaffolds allow the creation of protein fusions or the addition of N- or C-terminal tags. For instance, the prototype BioScaffold shown here can also be used to add N-terminal tags, as was demonstrated here through the introduction of a MBP fusion upstream of GFP. Another advantage of BioScaffolds is that a wide variety of parts can be introduced at a specific position in a single reaction, providing an approach to perform directed evolution and selection of circuits using BioBrick standard biological parts. Thus, BioScaffolds provide a compelling tool to extend idempotent assembly techniques, such as BioBrick assembly, and can even be utilized in combination with PCR based circuit assembly techniques.

## Methods

### Construction of prototype BioScaffold: part BBa_J70399

The prototype BioScaffold (BBa_J70399, the part number assigned by the Registry of Standard Biological Parts at [42]) was created using the RFP production circuit (BBa_J04450) as a template and the primers J70399-f (5'-gtttcttcgaattcgcggccgcttctagagatacatgaacatgcaatacgcaaacc-3') and J70399-r (5'-gtttcttcctgcagcggccgctactagtagagagcgttcaccgacaaacaacag-3'). Each primer contains a recognition site for the Type IIB REase PsrI as well as the standard BioBrick ends. The reactions contained 45 μl PCR SuperMix High Fidelity (Invitrogen), 12.5 pmoles of forward and reverse primer, and 1 ng template DNA in a 50 μl total volume. The PCR steps included a denaturation step of 96°C for 4 minutes followed by 36 cycles of a 94°C denaturation step for 30 seconds, a 52.3°C anneal step for 30 seconds, and a 68°C extend step for 2.5 minutes. Finally, the reactions were incubated at 68°C for 10 minutes before being cooled to 4°C until the reactions were halted. The samples and 1 μg of 2-log DNA ladder (New England Biolabs, Inc.) were electrophoresed in separate lanes on a 1% agarose gel. Sample bands of length 1000 base pairs were excised and purified with a QIAEX II Gel Extraction Kit (QIAGEN). The amplified linear DNA fragment was cloned into the BioBrick vector pSB1AT3, by digesting both the fragment and the vector with XbaI and PstI and performing the ligation using protocols adapted from BioBricks assembly kit (New England Biolabs, Inc.) to place the fragment into the vector. The ligation mixture was transformed into chemically competent *E. coli *strain TOP10 (Invitrogen) [12] and plated on Luria-Bertani (LB) agar plates supplemented with 15 μg/ml tetracycline and 100 μg/ml ampicillin.

### Assembly of the BioScaffold test circuit: part BBa_J70423

The test circuit consists of a promoter, the BioScaffold, and GFP assembled into the circuit "promoter-scar-BioScaffold-scar-GFP." The assembly was performed in two rounds using the BioBrick Assembly kit (New England Biolabs, Inc.) for three antibiotic (3A) assembly. First, the promoter (BBa_R0010 in BioBrick vector pSB1A2), the BioScaffold part (BBa_J70399 in BioBrick vector pSB1AT3), and the destination vector pSB1AK3 were digested. The upstream part, the promoter (BBa_R0010), was digested with EcoRI and SpeI. The downstream part, the BioScaffold part (BBa_J70399), was digested with XbaI and PstI. The destination vector pSB1AK3 was disgested with EcoRI and PstI. The digests were mixed together and ligated with T4 ligase, to form a composite part (BBa_J70405 in the vector pSB1AK3). The ligation mixture was transformed into chemically competent TOP10 cells as above and plated on LB agar plates containing 100 μg/ml ampicillin and 50 μg/ml kanamycin. 5 colonies that appeared red were verified by colony PCR and sequencing as described below.

Next, the composite part (BBa_J70405 in BioBrick vector pSB1AK3), the GFP (BBa_E0040 in BioBrick vector pSB1A2), and the destination plasmid pSB1AC3 were digested and ligated in the same manner as above, where BBa_J70405 was the upstream part and BBa_E0040 was the downstream part, to form the test circuit composite part (BBa_J70423). The ligation mixture was transformed into chemically competent TOP10 cells as above and plated on LB agar plates containing 100 μg/ml ampicillin and 35 μg/ml chloramphenicol. 5 colonies that appeared red were verified by colony PCR and sequencing as described below.

### Design of RBSs

The RBS calculator [71] was used to design the RBS sequences. The downstream GFP (BBa_E0040) was entered as the protein coding sequence. TACTAGAG was used as the presequence. Five different target translation initiation rates (10, 100, 1,000, 10,000, and 100,000) were entered into the calculator to generate the five different RBS sequences. The sequences generated by the calculator (see Table 1) were then utilized to create forward and reverse oligonucleotides, taking into account the cutting profile of the BioScaffold. The forward oligonucleotide has the sequence "G-RBS sequence-ATGCGTAAA" and the reverse oligonucleotide has the sequence reverse complement of "CTAGAG-RBS sequence-ATGC." After the replacement of the BioScaffold with the annealed oligonucleotides, the downstream scar disappears, while the upstream scar remains.

**Table 1:** Sequences generated by the RBS Calculator

Target	Rate	Sequence
10	10.5	CTAAATAGGAGGCTGGGAGTTCAACGAAACCCCT
100	95.6	CCCCCGTTCACTATACCGCAGGCCTTCTTTACAAA
1,000	950.1	ACATTAACCTACAAAGAACGTCGCAGAGGGA
10,000	9,953.7	TGTCGCGGATACTGATCCATAAAGGCCGGGGTT
100,000	94,459.0	TAGAGCCGTTAAAGAAGCTAGGAGGCCGAA

10, 100, 1,000, 10,000, and 100,000 were entered as target initiation rates in the RBS calculator. The RBS calculator uses a relative scale to relate relative translation initiation rate with five terms that quantify the strengths of molecular interactions involved in this process, with the result that any RBS sequence designed by the RBS Calculator can be related on this scale (further descriptions of this unitless parameter are available in the document [36] and FAQs [77,78] describing this software. Qualitatively from visual analysis of GFP expression in our samples and quantitatively based on our FACS results, we consider 10-100 to be low rates of translation initiation, 1,000 to be a medium rate, and 10,000 and above to be high rates of translation initiation.

### Design of the RBS-MBP-GS insert

The RBS-MBP-GS insert was created using the plasmid pMAL-p5e (New England Biolabs) as a template and the primers RBS-MBP-GS-GFPstart-f (5'-gaattcgcggccgcttctagagacgaacgctctctactagatgcctagagtcgccccctaagggcggaggtaggagaaactcaaatg aaaatcgaagaaggtaaactggtaatctg -3') and RBS-MBP-GS-GFPstart-r (5'-ctgcagcggccgctactagtagtaatatatgttcgatagattttacgagaaccagtctgcgcgtctttcagg -3') using the same PCR and gel extraction protocol as for BBa_J70399.

### Excision of BioScaffold part from the test circuit and replacement with RBSs

The oligonucleotides containing the RBSs were prepared by combining 8 μl of 100 μM forward oligonucleotide, 8 μl of 100 μM reverse oligonucleotide, 10 μl annealing buffer (100 mM Tris HCl pH 7.5, 1 M NaCl, 10 mM EDTA), and 74 μl milliQ water. They were then annealed by heating to 95°C for 2 minutes, ramping from 95°C to 25°C over 45 minutes, and then cooling to 4°C. We diluted 10 μl of oligonucleotides into a final volume of 1000 μl.

We digested the test circuit by combining 100 ng DNA, 1× Buffer Y (SibEnzyme, Inc.), 100 μg/ml Bovine Serum Albumin (SibEnzyme, Inc.), and 0.5 μL PsrI (SibEnzyme, Inc.) into a final volume of 50 μl. The restriction digest reaction was incubated for 1 hour at 30°C followed by 20 minutes at 65°C. In our experience digestion of more DNA dramatically increases the number of undigested products yielding red colonies upon transformation.

We performed the ligation by combining 5 μL of the PsrI digestion reaction (10 ng DNA), 0.2 μL of the diluted annealed oligonucleotides, 1× T4 DNA ligase reaction buffer, 200 units of T4 DNA ligase into a 20 μL total volume and cooling to 18°C for 30 minutes. The ligation mixture was transformed into chemically competent TOP10 cells as above and plated on LB agar plates containing100 μg/ml ampicillin and 35 μg/ml chloramphenicol. 5 non-red colonies per reaction were verified by colony PCR and sequencing as described below.

### Replacement of the BioScaffold with the RBS-MBP-GS insert to create BBa_J70631

Both the test circuit and the gel purified RBS-MBP-GS insert were digested with PsrI as described above in separate reactions. We performed the ligation by combining 5 μl of the test circuit digestion reaction, 5 μl of the RBS-MBP-GS insert digestion reaction, 1× T4 DNA ligase reaction buffer, 200 units of T4 DNA ligase into a 20 μl total volume and cooling to 18°C for 30 minutes. The transformation and sequencing of BBa_J70631 occurred as described previously for a test circuit containing an insert.

### Verification with colony PCR and sequencing

Colony PCR and subsequent gel electrophoresis were performed according to [12] except that the BioBrick primers BioBrick-f (BBa_G1004) and BioBrick-r (BBa_G1005) were used. The PCR protocol was the same as described for the BioScaffold part above, except that the extension step at 68°C occurred for 3.5 minutes instead of 2.5 minutes. The Massachusetts Institute of Technology Biopolymers Laboratory performed DNA sequencing, using the verification primers VF2 (BBa_G00100) and VR (BBa_G00101).

### Flow cytometry measurement of final circuit fluorescent intensity

Expression data were collected using a Becton-Dickinson FACSCAN flow cytometer with a 488-nm argon excitation laser and a 515-545 excitation filter [72]. Cells were grown in M9 media [73] (1× M9 salts, 2 mM MgSO4, 0.5% glycerol, 0.2% casamino acids, 2 mM thiamine) with ampicillin and chloramphenicol. The cultures were grown according to [74], except that the samples were measured in M9 medium [73].

### Purification of the MBP-GS-GFP protein using an amylose column

The BBa_J70631 construct, which contains a MBP-GS-GFP expression circuit, was expressed in *E. coli *Top10. The culture was grown overnight at 37°C in rich media (10 g typtone, 5 g yeast extract, 5 g NaCl, 2 g glucose) containing 100 μg/ml ampicillin and 35 μg/ml chloramphenicol, expanded 1:100 the next day and grown for 3 hours after the OD_600 _reached 0.6. The BBa_J04430 construct, which contains a GFP expression circuit, was grown in the same manner except the media did not contain chloramphenicol. Purification of a 40 ml culture of each construct was performed according to [75] using amylose resin (New England Biolabs), expect that the GFP and MBP-GS-GFP lysates were diluted until they exhibited equivalent fluorescent intensity and an equivalent volume of each cleared lysate (containing less MBP than the binding capacity of the amylose resin) was applied to each column.

### SDS-PAGE of MBP-GS-GFP

Samples were run on a 4-12% Bis-Tris gel (Invitrogen) and stained with Coomassie blue or SimplyBlue (Invitrogen) stain [76]. The contrast of the image was adjusted uniformly in Adobe Photoshop to simplify the visualization of the bands.

### Mass spectrometry of MBP-GS-GFP

The lanes from a SimplyBlue stained SDS PAGE gel were excised and submitted for analysis to the Proteomics Core Facility of the Koch Institute for Integrative Cancer Research at MIT. The gel bands were subjected to in-gel protein digestion with trypsin, following standard protocols. LC-MS/MS analyses were carried out using a nanoflow reversed phase HPLC (Agilent) and an LTQ ion trap mass spectrometer (Thermo Electron). Protein identifications were carried out by database search using Sequest software (Thermo Electron) against an *E. coli *protein database, generated from the Uniref100 protein database. The protein sequence of the MBP-GS-GFP fusion protein was added to the *E. coli *protein database.

### Identity of the bands at ~67 and ~134 kDa

Proteins in the bands at ~67 and ~134 kDa were identified by in-gel digestion and LC-MS/MS analysis.

## Competing interests

The authors declare that they have no competing interests.

## Authors' contributions

JEN, RD, and TFK conceived of and developed the idea of using BioScaffolds for circuit optimization (i.e., creating the biological equivalent of electronic integrated circuits.) JEN, RD, AMB, AEL, and TFK developed the idea of using BioScaffolds to create protein fusions using BioBrick standard biological parts rather than new assembly standards. JEN, AMB, and TFK conceived of and developed the initial BioScaffold standard. JEN, AMB, and AEL conceived of and developed the idea of using visual screening to distinguish properly excised BioScaffolds from BioScaffolds that remained in the circuit. JEN, SG, and TFK conceived of and JEN and SG performed the flow cytometry experiments. JEN and KAD conceived of and created the BioScaffold prototype, performed the assemblies, and excised and replaced the BioScaffold with RBSs. JEN, RD, SG, KAD, AMB, AEL, and TFK drafted the manuscript. All authors read and approved the final manuscript.

## Supplementary Material

Additional file 1 **Table S1 - BioScaffold designs for maximal excision (see additional file)**. Several Type IIB enzyme recognition sites are aligned to the scar sequence TACTAGAK to determine maximum excision to the left and to the right of the BioScaffold. The alignment of the recognition sites to the scar fixes the sequence at the start and end of the BioScafffold. We include several notes to clarify the table. First, the enzymes shown cut on both sides of their recognition site, not just one. For example, the cut sites and recognition sequence for PsrI is (7/12)GAACNNNNNNTAC(12/7) [64]. Second, the K (in the scar sequence TACTAGAK) is T for any protein coding region or other sequence that starts with ATG (i.e., TACTAGATG) and G for any other sequence. M represents A or C, R represents G or A, and Y represents C or T [60]. Third, recognition sequences for the enzyme are highlighted in **bold font**. Fourth, the internal cuts sites within the BioScaffold are not shown and the selection marker between the two recognition sites is represented as |...| In the prototype BioScaffold, the selection marker is a RFP reporter circuit. Fifth, the notation represents the location of the cut sites in condensed form. BioScaffold {w,x;y,z} notation is described in the Results section of the paper. Click here for file
