# Supplementary material for: Introduction of customized inserts for streamlined assembly and optimization of BioBrick synthetic genetic circuits
Source: J Biol Eng. 2010 Dec 20;4:17. doi: 10.1186/1754-1611-4-17 (PMC3022552; doi:10.1186/1754-1611-4-17)
Supplement: Additional file 1 — Table S1 - BioScaffold designs for maximal excision (see additional file). Several Type IIB enzyme recognition sites are aligned to the scar sequence TACTAGAK to determine maximum excision to the left and to the right of the BioScaffold. The alignment of the recognition sites to the scar fixes the sequence at the start and end of the BioScafffold. We include several notes to clarify the table. First, the enzymes shown cut on both sides of their recognition site, not just one. For example, the cut sites and recognition sequence for PsrI is (7/12)GAACNNNNNNTAC(12/7) [64]. Second, the K (in the scar sequence TACTAGAK) is T for any protein coding region or other sequence that starts with ATG (i.e., TACTAGATG) and G for any other sequence. M represents A or C, R represents G or A, and Y represents C or T [60]. Third, recognition sequences for the enzyme are highlighted in bold font. Fourth, the internal cuts sites within the BioScaffold are not shown and the selection marker between the two recognition sites is represented as |...| In the prototype BioScaffold, the selection marker is a RFP reporter circuit. Fifth, the notation represents the location of the cut sites in condensed form. BioScaffold {w,x;y,z} notation is described in the Results section of the paper. [file 1754-1611-4-17-S1.PDF]

Enzyme	Cut site and upstream scar	BioScaffold start	Selection Marker	BioScaffold end	Downstream scar and cut site	Notation
**AlfI**	↓NNNTACTAGA**G**<br>↑NNNNNATGATCT**C**	**CANNNNNNTGC**<br>**GTNNNNNNACG**	— . . . . —<br>— . . . . —	**GCANNNNNNTGC**<br>**CGTNNNNNNACG**	TACTAGAKNNNN↓<br>ATGATCTMNN↑	{10,12;12,10}
**ArsI**	↓NTACTAGA**G**<br>↑NNNNNNATGATCT**C**	**ACNNNNNNTTYG**<br>**TGNNNNNNAARC**	— . . . . —<br>— . . . . —	**CRAANNNNNNGTC**<br>**GYTTNNNNNNCAG**	TACTAGAKNNNNN↓<br>ATGATCTM↑	{8,13;13,8}
**BaeI**	↓NNNNNNNNNT**ACTAGAG**<br>↑NNNNNNNNNNNNNNA**TGATCTC**	**TAYC**<br>**ATRG**	— . . . . —<br>— . . . . —	**GRTACNNNNG**<br>**CYATGNNNNC**	**T**ACTAGAKNNNNNNNN↓<br>**A**TGATCTMNNN↑	{16,21;16,11}
**BarI**	↓TACTAGA**G**<br>↑NNNNNATGATCT**C**	**AAGNNNNNNTAC**<br>**TTCNNNNNNATG**	— . . . . —<br>— . . . . —	**GAAGNNNNNN**<br>**CTTCNNNNNN**	**TAC**TAGAKNNNNNNN↓<br>**ATG**ATCTMNN↑	{7,12;15,10}
**BcgI**	↓NNNTACTAGA**G**<br>↑NNNNNATGATCT**C**	**CANNNNNNTCG**<br>**GTNNNNNNAGC**	— . . . . —<br>— . . . . —	**GCANNNNNNTCG**<br>**CGTNNNNNNAGC**	TACTAGAKNNNN↓<br>ATGATCTMNN↑	{10,12;12,10}
**BdaI**	↓NNTACTAGAG<br>↑NNNNATGATCTC	**TGANNNNNNTCA**<br>**ACTNNNNNNAGT**	— . . . . —<br>— . . . . —	**TGANNNNNNTCA**<br>**ACTNNNNNNAGT**	TACTAGAKNNNN↓<br>ATGATCTMNN↑	{9,11;12,10}
**BplI**	↓NNNTACTA**GAG**<br>↑NNNNNNNNATGAT**CTC**	**NNNNNCTC**<br>**NNNNNGAG**	— . . . . —<br>— . . . . —	**GAGNNNNNCTC**<br>**CTCNNNNNGAG**	TACTAGAKNNNNN↓<br>ATGATCTM↑	{10,15;13,8}
**BsaXI**	↓NNNNNNNNT**ACTAGAG**<br>↑NNNNNNNNNNNA**TGATCTC**	**CTCC**<br>**GAGG**	— . . . . —<br>— . . . . —	**GGAGNNNNNG**<br>**CCTCNNNNNC**	**T**ACTAGAKNNNNN↓<br>**A**TGATCTMNN↑	{15,18;13,10}
**CspCI**	↓NNNTACTAGAG<br>↑NNNNNATGATCTC	**CAANNNNNGTGG**<br>**GTTNNNNNCACC**	— . . . . —<br>— . . . . —	**CCACNNNNNTTG**<br>**GGTGNNNNNAAC**	TACTAGAKNNNNN↓<br>ATGATCTMNNN↑	{10,12;13,11}
**FalI**	↓TACTAGAG<br>↑NNNNNATGATCTC	**AAGNNNNNCTT**<br>**TTCNNNNNGAA**	— . . . . —<br>— . . . . —	**AAGNNNNNCT**<br>**TTCNNNNNGA**	**T**ACTAGAKNNNNNN↓<br>**A**TGATCTMN↑	{7,12;14,9}
**PpiI**	↓NNNTACTA**GAG**<br>↑NNNNNNNNATGAT**CTC**	**NNNNNCTTC**<br>**NNNNNGAAG**	— . . . . —<br>— . . . . —	**GAACNNNNNCTC**<br>**CTTGNNNNNGAG**	TACTAGAKNNNNN↓<br>ATGATCTM↑	{10,15;13,8}
**PsrI**	↓TACTAGA**G**<br>↑NNNNNATGATCT**C**	**AACNNNNNNTAC**<br>**TTGNNNNNNATG**	— . . . . —<br>— . . . . —	**GAACNNNNNN**<br>**CTTGNNNNNN**	**TAC**TAGAKNNNNNNN↓<br>**ATG**ATCTMNN↑	{7,12;15,10}
**TstI**	↓TACTAGA**G**<br>↑NNNNNATGATCT**C**	**GANNNNNNGTG**<br>**CTNNNNNNCAC**	— . . . . —<br>— . . . . —	**GGANNNNNNGTG**<br>**CCTNNNNNNCAC**	TACTAGAKNNNNN↓<br>ATGATCTM↑	{7,12;13,8}
